# Small bowel gastrointestinal stromal tumor presenting with involvement of the adjacent small bowel and hemoperitoneum case report

**DOI:** 10.1016/j.amsu.2020.08.024

**Published:** 2020-09-03

**Authors:** Bhavana Makkapati, Atul K. Nanda

**Affiliations:** a Medical Student, St. George's University School of Medicine, USA; b Chairman of Surgery, Norwegian American Hospital, Chicago, IL, USA

**Keywords:** Case report, General surgery, GI, Pathology, Oncology

## Abstract

Accounting for less than one percent of all GI tumors, gastrointestinal stromal tumors are uncommon neoplasms that arise from the intestinal cells of Cajal. They can arise anywhere along the gastrointestinal tract, but most often arise from the stomach or small bowel. Usually, they are small and present with vague symptoms such as abdominal pain and are found incidentally. They usually don't involve other structures unless they metastasize. Here, we present a case of small bowel GIST. The patient is a 72-year-old male who was seen in the clinic with symptoms of abdominal pain for 4 months along with intermittent gastrointestinal bleeding for 6 years. Imaging studies revealed a mass in the small bowel with no signs of obstruction. During surgery, a 9 cm by 9 cm small bowel mass was identified with adhesions to the appendix, omentum, and a segment of small bowel. There was also a hemoperitoneum. En bloc resection was performed with clean margins. Histopathology report showed malignant gastrointestinal stromal tumor with focal involvement of adjacent small bowel loop.

## Highlights

• GIST's usually don't involve other structures unless they metastasize.
• 20–30% of GIST's involve the small bowel and exhibit an endophytic growth pattern.
• GIST small bowel tumors with adjacent small bowel involvement and pseudocapsule is very rare.
• Surgery is the best treatment option especially for removal of pseudocapsule without rupture to prevent seeding.

## 1 Introduction

Gastrointestinal Stromal Tumor (GIST) originates from the intestinal cells of Cajal, which constitute the autonomic nervous system of the intestine [1,11]. GIST's are the most common mesenchymal tumor of the intestinal tract but account for less than 1% of all tumors within the gastrointestinal system [2]. Majority of these tumors are benign but a small percentage of them have malignant potential, especially intestinal GISTs [3]. GISTs can occur anywhere from the esophagus to the rectum. However, 60–70% arise from the stomach, 20–30% from the small bowel, and less than 10% elsewhere in the GI tract or extraintestinal sites [3]. They usually have an endophytic growth pattern with the tumor arising from the muscularis mucosa or propria layers of the GI tract [5]. All of the following work is reported in line with SCARE criteria [19].

Estimated incidence of GIST tumors is 10–20 cases per million, with the majority of cases in patients over 50 and mean age of diagnosis being around 64 years of age [[2], [3], [4]]. GIST tumors usually present with a wide array of symptoms. In general, most cases are found incidentally during another procedure being done [2]. About 28% of GIST tumors arising from the small intestine and 50% arising from the stomach present with overt or occult GI bleeding [2]. Another 8–17% present with abdominal pain as seen in the following case [2].

### 1.1 Case

**Image 1:**
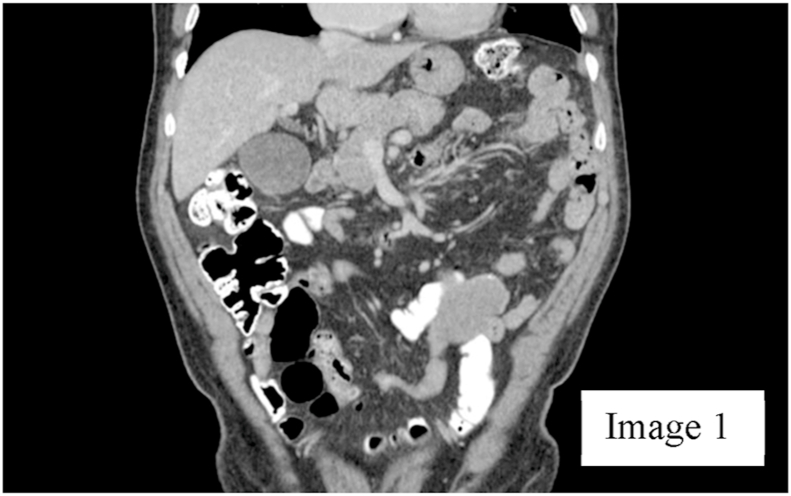
Coronal plane of CT scan of abdomen.

**Image 2:**
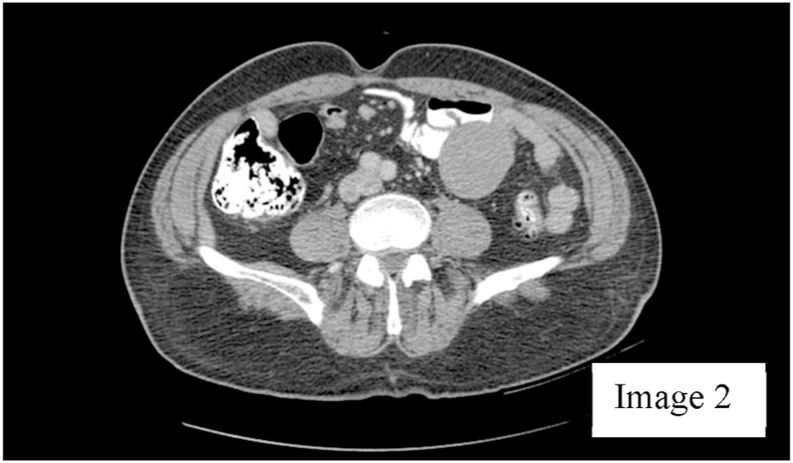
Axial plane of CT scan of abdomen.

A 72-year-old male presented to the clinic with generalized abdominal pain for 4 months that was constant and dull. Patient had a history of intermittent GI bleeding for about 6 years with no known source of bleeding despite having a workup at different hospitals. Past history includes hypertension, benign prostatic hyperplasia, gastritis, and anemia. Patient had undergone colonoscopy and esophagogastroduodenoscopy about four months ago that showed no abnormalities. He subsequently underwent a computed tomography scan of abdomen that showed a lobulated mass like structure originating from a bowel anastomosis. However, the patient did not give a history of any abdominal surgery, nor did he have any scars on his abdomen to indicate any previous abdominal surgery. On reviewing the CT scan, there was a linear calcification inside the mass, which seemed to arise from the small bowel or its mesentery without evidence of bowel obstruction Image 1, Image 2.

**Image 3:**
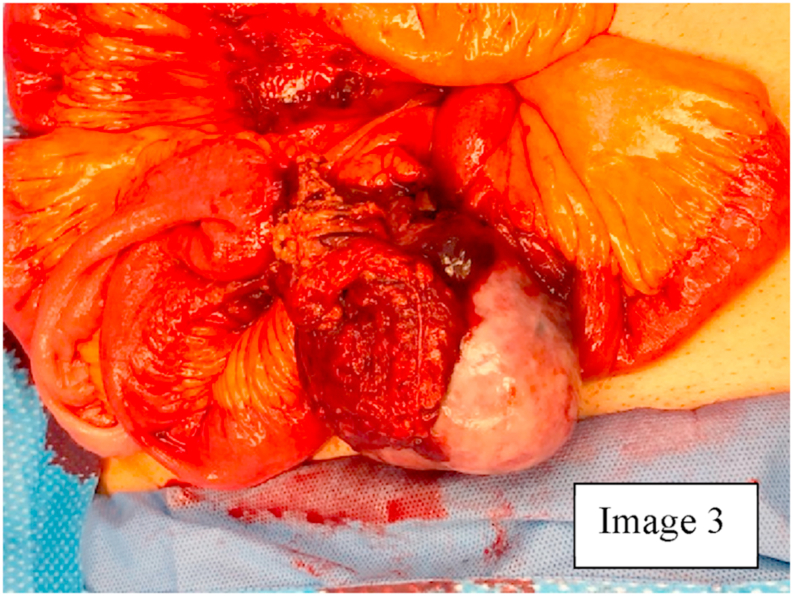
GIST adhering to small bowel loops and omentum.

**Image 4:**
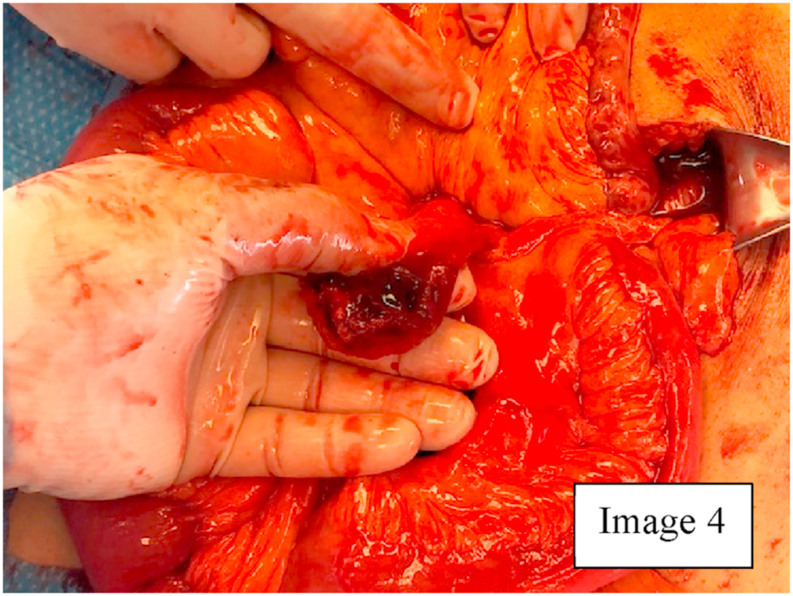
Small bowel mass with defect in capsule.

**Image 5:**
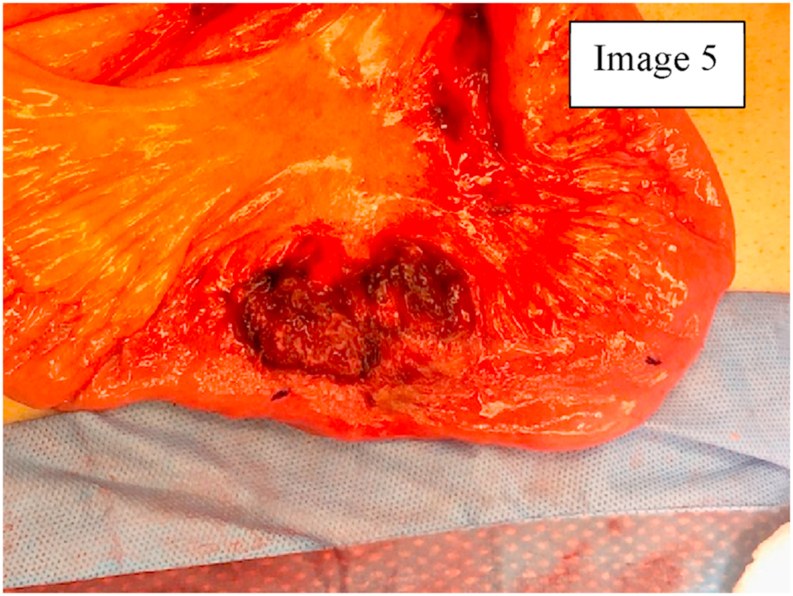
Pseudocapsule adhered to small bowel serosa.

Due to the findings on CT scan, a diagnostic laparoscopy was planned with biopsy/resection of the mass. During diagnostic laparoscopy, there was hemoperitoneum in the pelvis and Morrison's pouch. On examination of the small bowel, the mass was located in the distal small bowel with omentum and loops of small bowel densely adhering to and covering the mass [Image 3]. Diagnostic laparoscopy was converted to a mini laparotomy due to size and complexity of the mass [14]. On exploration and removal of the superficial adhesions with small bowel loops, the omentum, small bowel mesentery, appendix, and another segment of small bowel about 1.5 feet from ileocecal junction were densely adhered to the solid mass with a defect in the capsule of the mass, that seemed to be the source of bleeding that had caused the hemoperitoneum [Image 4]. An intraoperative frozen section of the mass showed spindle cells that indicated GIST. The pseudocapsule of the mass was adhered to the serosa of the small bowel and appendix Image 4, Image 5. Therefore, the patient underwent partial omentectomy, resection of segment of small bowel with the adhered pseudocapsule and appendectomy. This was done to ensure excision of the tumor with adequate margins. There were no enlarged mesenteric lymph nodes, liver masses, omental or peritoneal nodules. Patient's postoperative course was uneventful, and he was discharged home on post-operative day four after tolerating a regular diet and having bowel function. Patient was seen in the clinic and was noted to have good healing of the incision.

**Image 6:**
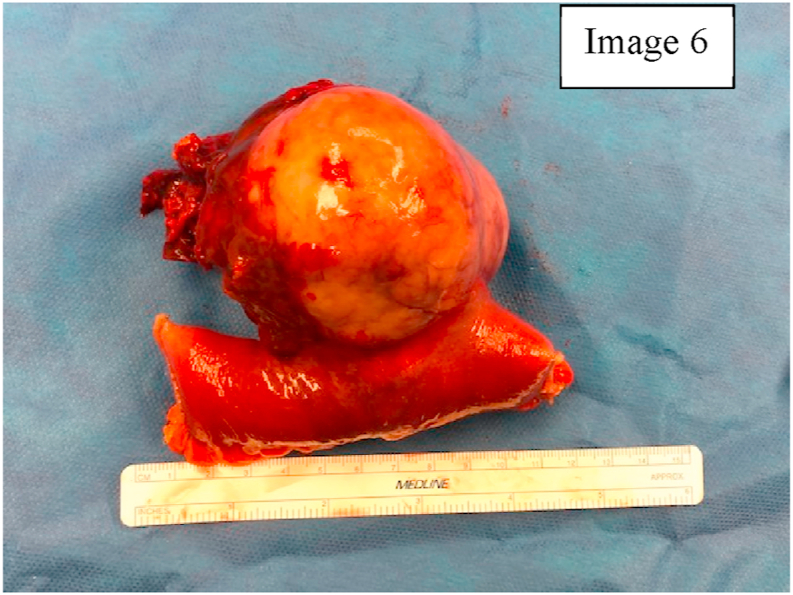
Excised Gastrointestinal Stromal Tumor.

Histopathological examination of mass showed a gastrointestinal stromal tumor measuring 9.5 × 9 × 8 cm [Image 6]. Mitoses were 10 per 50 high power fields. There was no lymphovascular infiltration or metastasis noted. Immunohistochemically, the tumor cells were positive for Ckit (CD117) Dog1, Vimentin and negative for CD34, Desmin, Chromogranin, and S100. According to TMN staging, the pathological stage of the excised tumor is PT3 Nx Mn/a, stage IIIB. These findings indicate a high risk of recurrence at both the one year and five-year mark [8,12]. Small bowel attached to the mass showed a 1mm area of tumor, appendix was negative for tumor. The margins of small bowel and appendix were free of tumor. The tumor is high risk due to pathological staging (Stage IIIB), rupture of pseudocapsule leading to hemoperitoneum and involvement of adjacent segments of small bowel. Patient would be receiving adjuvant imatinib mesylate therapy, a selective tyrosine kinase inhibitor and would be under close surveillance for recurrence [13].

## 2 Discussion

Gastrointestinal stromal tumors account for less than one percent of all GI tumors and have a wide array of presenting symptoms ranging from asymptomatic to bowel obstruction. Sixty to seventy percent of GIST's are found in the stomach as the primary site, with the other twenty to thirty percent found in the small bowel, and less than ten percent from elsewhere in the GI tract [2]. They usually exhibit an endophytic growth pattern involving the muscularis mucosa or propria layers and very rarely show exophytic growth [5]. GIST's involving the small bowel, of a size greater than 5cm, have a high incidence of bleeding, as seen in our case with the patient having undergone workup multiple times at different hospitals for 6 years with no diagnosis [16]. Increased bleeding from small bowel GIST is due to the decreased stromal collagen leading to thin walled vessels that have a higher chance of hemorrhage or hemoperitoneum as in our case [16]. Therefore, once other diagnosis are ruled out especially with tests such as endoscopy, it is prudent to consider GIST's, especially if there is a high index of clinical suspicion.

There is no specific diagnostic test that is very sensitive or specific for detection of GIST. A computed tomography scan, magnetic resonance imaging, upper endoscopy are some of the tests used based off of clinical suspicion with CT scans being the most effective [2]. CT scan of our patient showed a mass involving the small bowel with linear calcifications and no bowel obstruction. Surgery is the best option for treatment as it allows for clear margins and longest survival chances [15]. During mini laparotomy a mass was removed with the surrounding pseudocapsule. Patient already had rupture of the pseudocapsule with hemoperitoneum. Rupture of the pseudocapsule leads to seeding and worsens prognosis [17]. Surgery also showed direct involvement of adjacent small bowel by primary tumor with adhesions to appendix, omentum, and mesentery. Involvement of other structures in the abdomen such as liver and peritoneum are due to metastatic spread of GIST hematogenously or through peritoneal seeding from a ruptured GIST capsule [18]. In spite of rupture of the capsule and hemoperitoneum, no peritoneal or omental metastasis/seeding were seen in our case. After surgery, adjuvant therapy with imatinib mesylate has proven to be effective in decreasing recurrence [5,9].

There are many ways to classify GIST's and the traditional TNM staging exists but is rarely used in clinical settings due to the fact that most GIST's aren't necessarily classified into benign and malignant [9]. Although it is used in pathology reports. A prognostic criterion proposed by the National Institutes of Health are used clinically which classify GIST's into very low, low, intermediate, and high risk for malignancy based on tumor size and mitotic rate [Table 1] [9]. These tumors rarely invade lymph nodes and rather spread hematogenously to the liver, bone, and lung [2,5]. They also spread by direct extension to surrounding structures within the abdominal cavity such as the peritoneum [5,10]. Most of these tumors are always positive for C-KIT and Dog1 with other variable markers such as S100, Desmin, CD34, and Vimentin may or may not be present [2,3]. In our case, the patient was positive for Ckit (CD117) Dog1, Vimentin.

**Table 1:** Proposed modification of NIH consensus criteria for risk stratification of GISTs.

Risk Group	Size (cm)	Mitotic Rate (per 50 HPF)
Original NIH Criteria		
Very low risk	<2	<5
Low risk	2 to 5	<5
Intermediate risk	<5	6 to 10
	5 to 10	<5
High Risk	>5	>5
	>10	Any
	Any	>10
**Proposed Criteria**		
Level I	≤5	<5
Level II	<5	6 to 10
	5 to 10	<5
Level III	≤5	>10
	5 to 10	6 to 10
	>10	<5
Level IV	>5	>10

NIH: National Institutes of Health; GIST: gastrointestinal stromal tumor; HPF: high-power fields.

* Mitotic rate is counted in an area of 5 square millimeters (㎟) on the glass slide section. For older microscopes with traditional field size optics, 50 HPF is equivalent to 5 ㎟. For modern microscopes with wider 40x lenses/fields, 20 HPF is equivalent to 5 ㎟. If necessary, the field of view should be measured to determine the actual number of HPF required to cover a 5 ㎟ area.

Large GIST tumors have a change of recurrence even after surgery though the rate of recurrence decreases if treated with imatinib mesylate. This case was of a rather large tumor that intraoperatively was seen to have dense adhesions to the adjacent small bowel, omentum, and appendix. These adhesions were so significant that partial omentectomy, two small bowel resections and anastomosis and appendectomy were done. There was also hemoperitoneum, which in our case seemed to be due to the size of the tumor causing hemorrhage in the tumor, thereby leading to rupture of the capsule.

## 3 Conclusion

Gastrointestinal stromal tumors originating from the small bowel are uncommon. Small bowel GIST with involvement of adjacent small bowel and rupture of the pseudocapsule leading to hemoperitoneum are even rare findings.

High index of suspicion is warranted in patients having recurrent GI bleeding without obvious source on esophagogastroduodenoscopy and colonoscopy. Further imaging modalities including CT scan and MRI of abdomen can help in making the diagnosis.

Recommended treatment of choice for small bowel GIST is en bloc surgical resection with inclusion of pseudocapsule. Radical lymphadenectomy is not routinely indicated as lymph node involvement is less than 10%. Treatment with selective tyrosine kinase inhibitor is recommended in high risk cases to reduce risk of recurrence.

### Consent

Written informed consent was obtained from the patient for publication of this case report and accompanying images. A copy of the written consent is available for review by the Editor-in-Chief of this journal on request.
